# 2801. Antimicrobial Spectrum of Plazomicin and Other Aminoglycosides against Multidrug-Resistant and Carbapenem-Resistant Enterobacterales When Applying New Breakpoints

**DOI:** 10.1093/ofid/ofad500.2412

**Published:** 2023-11-27

**Authors:** Helio S Sader, Rodrigo E Mendes, S J Ryan Arends, Cecilia G Carvalhaes, Mariana Castanheira

**Affiliations:** JMI Laboratories, North Liberty, Iowa; JMI Laboratories, North Liberty, Iowa; JMI Laboratories, North Liberty, Iowa; JMI Laboratories, North Liberty, Iowa; JMI Laboratories, North Liberty, Iowa

## Abstract

**Background:**

In March 2023, the Clinical and Laboratory Standards Institute (CLSI) lowered the Enterobacterales susceptible (S)/resistant breakpoints for amikacin from ≤16/≥64 mg/L to ≤4/≥16 mg/L and gentamicin and tobramycin from ≤4/≥16 mg/L to ≤2/≥8 mg/L. As aminoglycosides are frequently used to treat infections caused by multidrug-resistant (MDR) and carbapenem-resistant Enterobacterales (CRE), we evaluated the activity of plazomicin and the impact of CLSI breakpoint changes on the susceptibility rates of Enterobacterales collected from US medical centers.

**Figure:**
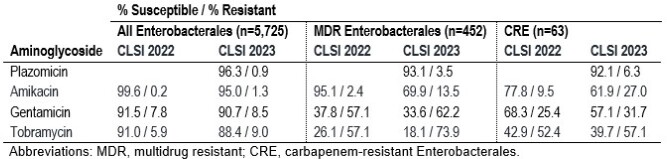

**Methods:**

5,725 Enterobacterales isolates were consecutively collected (1/patient) from 36 US medical centers in 2020–2022 and susceptibility tested by broth microdilution against amikacin, gentamicin, tobramycin, and plazomicin. Susceptibility rates were calculated using both current CLSI/USFDA breakpoints and the recently revised CLSI breakpoints. Aminoglycoside-nonsusceptible isolates were screened for genes encoding aminoglycoside-modifying enzymes (AMEs) and 16S rRNA methyltransferases (16RMT).

**Results:**

Plazomicin was active against 96.3% of isolates and retained potent activity against MDR (93.1%S) and CRE (92.1%S) isolates. The highest variations in susceptibility rates due to breakpoint changes were observed with amikacin, especially among MDR (95.1%S to 69.9%S; Table) and CRE (77.8%S to 61.9%S). Against all Enterobacterales, amikacin susceptibility rates decreased from 99.6%S to 95.0%S; susceptibility to gentamicin and tobramycin decreased 0.8% and 2.6%, respectively. Gentamicin and tobramycin showed limited activity against MDR and CRE with both 2022 and 2023 breakpoints. AME-encoding genes were observed in 474 (8.3% of isolates) and 16RMT was found in 7 isolates (0.1%). Plazomicin was active against 97.3% of AME producers.

**Conclusion:**

Amikacin’s spectrum of activity against CRE and MDR Enterobacterales was drastically reduced when interpretative criteria based on PK/PD parameters currently used to establish breakpoints to other antimicrobials were applied. Plazomicin is markedly more active than amikacin, gentamicin, or tobramycin against CRE and MDR Enterobacterales causing infections in US medical centers.

**Disclosures:**

**Helio S. Sader, MD, PhD, FIDSA**, AbbVie: Grant/Research Support|Basilea: Grant/Research Support|Cipla: Grant/Research Support|Paratek: Grant/Research Support|Pfizer: Grant/Research Support|Shionogi: Grant/Research Support **Rodrigo E. Mendes, PhD**, AbbVie: Grant/Research Support|Basilea: Grant/Research Support|Cipla: Grant/Research Support|Entasis: Grant/Research Support|GSK: Grant/Research Support|Paratek: Grant/Research Support|Pfizer: Grant/Research Support|Shionogi: Grant/Research Support **S J Ryan Arends, PhD**, Cipla: Grant/Research Support|GSK: Grant/Research Support **Cecilia G. Carvalhaes, MD, PhD**, AbbVie: Grant/Research Support|bioMerieux: Grant/Research Support|Cipla: Grant/Research Support|CorMedix: Grant/Research Support|Melinta: Grant/Research Support|Pfizer: Grant/Research Support **Mariana Castanheira, PhD**, AbbVie: Grant/Research Support|Basilea: Grant/Research Support|bioMerieux: Grant/Research Support|Cipla: Grant/Research Support|CorMedix: Grant/Research Support|Entasis: Grant/Research Support|Melinta: Grant/Research Support|Paratek: Grant/Research Support|Pfizer: Grant/Research Support|Shionogi: Grant/Research Support

